# Genome-Wide Characterization of TCP Transcription Factors in Common Glasswort (*Salicornia europaea*) and Their Expression Analysis Under Salt Stress

**DOI:** 10.3390/ijms27125514

**Published:** 2026-06-18

**Authors:** Liuhan Wu, Shuqi Yang, Fang Wang, Wenqi Yang, Chijie Yin, Zexuan Hao, Zhiyong Wang, Rundong Jia, Meiling Fu, Shaojun Wu, Boping Tang, Yuan Qin, Yan Cheng, Gang Wang

**Affiliations:** 1 Fujian Provincial Key Laboratory of Haixia Plant Systems Biology, School of Future Technology, College of Plant Protection, Fujian Agriculture and Forestry University, Fuzhou 350002, China; 2 Jiangsu Provincial Key Laboratory of Coastal Wetland Bioresources and Environmental Protection, Jiangsu Synthetic Innovation Center for Coastal Bioagriculture, Yancheng Teachers University, Yancheng 224007, China

**Keywords:** *Salicornia europaea*, TCP, salt stress, genome-wide analysis, halophyte

## Abstract

TEOSINTE BRANCHED1/CYCLOIDEA/PROLIFERATING CELL FACTOR (TCP) are plant-specific regulators involved in growth, development, and responses to abiotic stresses, yet their roles in halophytes remain largely unexplored. In this study, we performed a genome-wide identification of TCP family members in the extreme halophyte *Salicornia europaea*, uncovering 15 non-redundant genes (*SeurTCPs*) classified into PCF, CIN, and CYC/TB1 subfamilies. Gene structure and conserved motif analyses revealed that *SeurTCPs* are largely intronless and maintain the canonical TCP domain, while showing subfamily-specific variations in motif composition and secondary/tertiary structures. Promoter analysis identified abundant stress and hormone-responsive *cis*-elements, particularly ABRE and STRE, suggesting potential involvement in salt stress signaling. Protein–protein interaction network prediction highlighted CIN and PCF members as hub nodes, indicating central roles in growth and stress response regulation. Quantitative Real-Time Reverse Transcription Polymerase Chain Reaction (qRT-PCR) analysis showed that most SeurTCP genes were responsive to salinity treatment, although the extent of transcriptional variation differed among subfamilies. Collectively, our results indicate that SeurTCPs balance conserved structural functions with subfamily-specific regulatory roles, contributing to *S. europaea* adaptation to extreme saline environments. This study provides valuable candidate genes for elucidating plant salt tolerance mechanisms and for potential crop improvement.

## 1. Introduction

Plants are continuously challenged by diverse environmental constraints during growth and development [1]. These stresses can be broadly classified into biotic stresses, such as pathogen infection and herbivory, and abiotic stresses, including salinity, drought, extreme temperature, nutrient imbalance, and heavy-metal toxicity [2]. Among abiotic factors, salt stress is one of the most severe limitations to plant growth, development, yield formation, and product quality [3]. Excessive salinity imposes osmotic stress and ion toxicity through the accumulation of Na^+^ and Cl^−^, disrupts cellular water status and nutrient uptake, impairs photosynthesis, accelerates reactive oxygen species (ROS) accumulation, and ultimately inhibits biomass accumulation and reproductive development. At the field scale, salinity also changes soil physicochemical properties, including salt accumulation, soil compaction, surface crust formation, and reduced soil water availability, thereby further constraining plant establishment and productivity [4,5]. To survive in long-term high-salinity environments, plants have evolved complex molecular, physiological, and biochemical mechanisms to maintain ion balance and normal development [6]. However, the core molecular networks governing these adaptive processes remain largely unclear.

Transcription factors (TFs) regulate gene expression by specifically binding to promoter regions of target genes and serve as key nodes in plant responses to abiotic stresses [7]. Abiotic stress responses are controlled by interconnected transcription factor networks rather than by a single transcription factor family. Several major transcription factor families, including MYB, NAC, WRKY, DREB/ERF, bZIP, and bHLH, have been reported to regulate stress-responsive genes and to participate in osmotic adjustment, antioxidant defense, abscisic acid (ABA) signaling, and downstream protective metabolism [8,9,10]. For example, the NAC transcription factor *FvNAC29* from *Fragaria vesca* enhanced salt and cold tolerance when overexpressed in *Arabidopsis thaliana*. The transgenic plants maintained higher proline and chlorophyll contents and higher activities of catalase, peroxidase, and superoxide dismutase under salt or low-temperature stress [11]. Similarly, *MbWRKY50* from *Malus baccata* improved cold and drought tolerance in transgenic tomatoes by enhancing antioxidant capacity and activating genes associated with ABA biosynthesis/signaling and the CBF/DREB pathway [12].

The TEOSINTE BRANCHED1/CYCLOIDEA/PROLIFERATING CELL FACTOR (TCP) family represents a plant-specific group of transcriptional regulators, named after the first identified members: Teosinte branched1 (*TB1*), Cycloidea (*CYC*), and Proliferating Cell Factor 1/2 (*PCF1/2*) [13,14,15]. Based on the structural features of the TCP domain, the family is typically divided into two major classes: Class I and Class II, with the latter further subdivided into CIN and CYC/TB1 clades [16,17]. Numerous studies have shown that TCP transcription factors play key roles in plant growth and development, including regulating seed germination, leaf morphogenesis, and hormone signaling [18,19,20]. In addition, TCP genes are also involved in responses to abiotic stresses; for example, *Arabidopsis TCP5* and *TCP17* enhance thermotolerance by regulating *PIF4* expression [21], while rice *OsPCF2*, *OsPCF5*, and *OsPCF6* participate in drought and cold stress responses [22].

Although the TCP gene family has been extensively studied in model plants regarding growth and developmental regulation, and evidence indicates their involvement in moderate abiotic stress responses, their roles in extreme salt environments remain poorly understood. In particular, in halophytes, the genome-wide evolution and transcriptional regulatory mechanisms by which TCP genes integrate developmental cues with high-salinity adaptation remain largely unexplored. The Common Glasswort (*Salicornia europaea*) is a typical extreme halophyte widely distributed in saline marshes and intertidal zones [23], capable of completing its life cycle under approximately 3% salinity [24]. It adapts to high-salinity environments through succulent leaves and efficient ion compartmentalization [25], and under long-term environmental selection, it has developed unique salt-tolerance metabolic and regulatory networks, making it an ideal system to study extreme salt adaptation in plants.

Based on this, the present study conducted a comprehensive genome-wide identification and systematic analysis of the TCP gene family in *S. europaea*, including physicochemical properties, chromosomal localization, phylogenetic relationships, gene structures, conserved motifs, *cis*-acting elements, collinearity patterns, and protein–protein interaction networks. Additionally, Quantitative Real-Time Reverse Transcription Polymerase Chain Reaction (qRT-PCR) was employed to assess their expression profiles under salt stress to identify key responsive genes. This study aims to reveal the evolutionary characteristics of the *S. europaea* TCP family and its potential regulatory roles in extreme salt adaptation, providing candidate genes for crop salt-tolerance improvement.

## 2. Results

### 2.1. Identification and Characterization of the TCP Gene Family in Salicornia europaea

**Figure 1:**
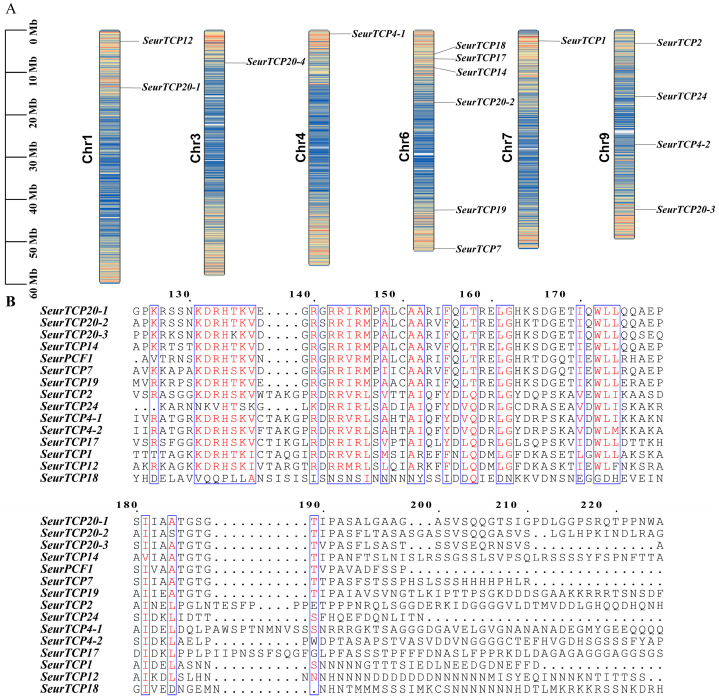
Chromosomal localization and multiple sequence alignment of SeurTCPs. (**A**) Distribution of SeurTCP genes on the chromosomes of *S. europaea*. The gradient color background on the chromosomes indicates gene density (from low in blue to high in red). The chromosome numbers are indicated on the left side of each chromosome. (**B**) Multiple sequence alignment of the conserved TCP domains in SeurTCP proteins. Identical and highly conserved amino acid residues are highlighted with red text and blue boxes.

Based on the whole-genome data of *S. europaea*, a genome-wide scan for TCP conserved domains (PF03634) was performed using HMMER 3.0, and BLASTp searches were conducted using *Arabidopsis* TCP protein sequences as queries. Candidate sequences were further validated for the presence of TCP domains using SMART and the NCBI Conserved Domain Database (CDD), resulting in the identification of 15 non-redundant TCP family members (*SeurTCPs*). According to the *Arabidopsis* TCP family classification and sequence homology analysis, the *S. europaea* TCP family can be divided into three subfamilies: PCF, CIN, and CYC/TB1. Chromosomal mapping indicated that the 15 SeurTCP genes are unevenly distributed across chromosomes 1, 3, 4, 6, 7, and 9, with the highest gene numbers located on chromosomes 6 and 9 (Figure 1A).

Physicochemical property analysis revealed that SeurTCP proteins range from 199 to 584 amino acids in length, with corresponding molecular weights of 21.96–63.69 kDa and pI ranging from 5.49 to 9.46, including both acidic and basic proteins (Table 1). All members exhibited negative GRAVY values (−1.251 to −0.496), indicating overall hydrophilicity. Notably, both sequence length and physicochemical properties showed substantial heterogeneity among family members.

Multiple sequence alignment revealed that all SeurTCP proteins contain a highly conserved TCP domain of approximately 60 amino acids (Figure 1B). The N-terminal basic region of this domain is rich in arginine and lysine residues, forming the core DNA-binding site and exhibiting a typical basic helix-loop-helix (bHLH-like) secondary structure. The helix II region contains conserved tryptophan and leucine residues, representing classical conserved sites, with highly conserved residues mainly distributed within the helical regions. Based on specific residue insertions or deletions within the TCP domain, the *S. europaea* TCP family can be further classified into Class I (PCF) and Class II (CIN + CYC/TB1). Among them, Class I members such as *SeurTCP20-1/2/3/4*, *SeurTCP7*, and *SeurTCP19* lack four specific amino acids in the TCP domain compared with Class II members and exhibit Class I-specific conservation at certain positions.

In summary, the *S. europaea* TCP family exhibits diversity in genomic distribution, physicochemical properties, and domain conservation, providing a foundation for further functional analyses of their roles in developmental regulation and salt stress responses.

### 2.2. Phylogenetic Relationships and Intra- and Inter-Species Collinearity Analysis of the SeurTCP Family

**Figure 2:**
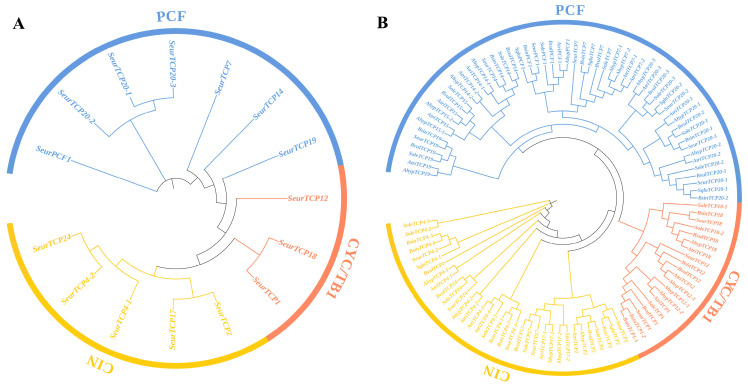
Phylogenetic relationships and multispecies synteny analysis of SeurTCPs. (**A**) Unrooted phylogenetic tree of SeurTCP proteins in *Salicornia europaea*. (**B**) Multispecies phylogenetic tree of TCP proteins from *S. europaea* and related species. In both (**A**,**B**), the outer rings are color-coded to distinguish different TCP subfamilies (PCF, CIN, and CYC/TB1).

Based on intra-species phylogenetic analysis of *S. europaea* (Figure 2A), the 15 SeurTCP members were clustered into three monophyletic clades corresponding to the classical PCF, CIN, and CYC/TB1 subfamilies. This classification indicates that the *S. europaea* TCP family retains the three core subfamilies observed in plant TCP families, largely consistent with classification frameworks reported in other plant species.

Cross-species comparison showed that TCP members from *S. europaea* and six closely related species, including *Su. glauca*, *Be. vulgaris*, and *Amaranthus* species, were strictly grouped into the same three independent clades in the evolutionary tree, with no observed inter-subfamily mixing (Figure 2B). This suggests that the three TCP subfamily lineages were already established before species divergence and exhibit high evolutionary conservation.

While the overall clade structure is preserved, species-specific subclade variations were observed. Intra-species analysis revealed that the PCF subfamily contains the largest number of members in *S. europaea* and forms distinct subgroups (e.g., *SeurTCP20-1/2/3*), indicating active expansion of this subfamily following species differentiation. Overall, the phylogenetic characteristics of SeurTCPs show a combination of “ancient clades” and “recent expansions”, and such conservation with partial redundancy may provide a genetic basis for functional diversification under extreme salt stress.

Intra-genomic collinearity analysis across chromosomes 1–9 revealed that TCP genes on Chr6 form clear collinear blocks with homologous genes on Chr1 and Chr9, suggesting that the family has undergone segmental duplication and tandem repeat-mediated expansion within the genome, reflecting the evolutionary conservation of the genomic regions harboring TCP genes (Figure S1).

Inter-species collinearity analysis indicated the presence of conserved collinear blocks between *S. europaea* and closely related species (e.g., *Su. glauca*, *Be. vulgaris*), as well as more distantly related species (e.g., *Am. hypochondriacus*, *Am. tricolor*) (Figure S2). Further analysis showed that both the number of collinear gene pairs and gene order consistency were higher between *S. europaea* and closely related species than with more distant species, indicating greater evolutionary conservation of the TCP family among closely related taxa. In contrast, disruption of collinear blocks and changes in gene order in distant species may reflect functional divergence of TCP genes during phylogenetic differentiation.

### 2.3. Gene Structure and Conserved Motifs of SeurTCPs

**Figure 3:**
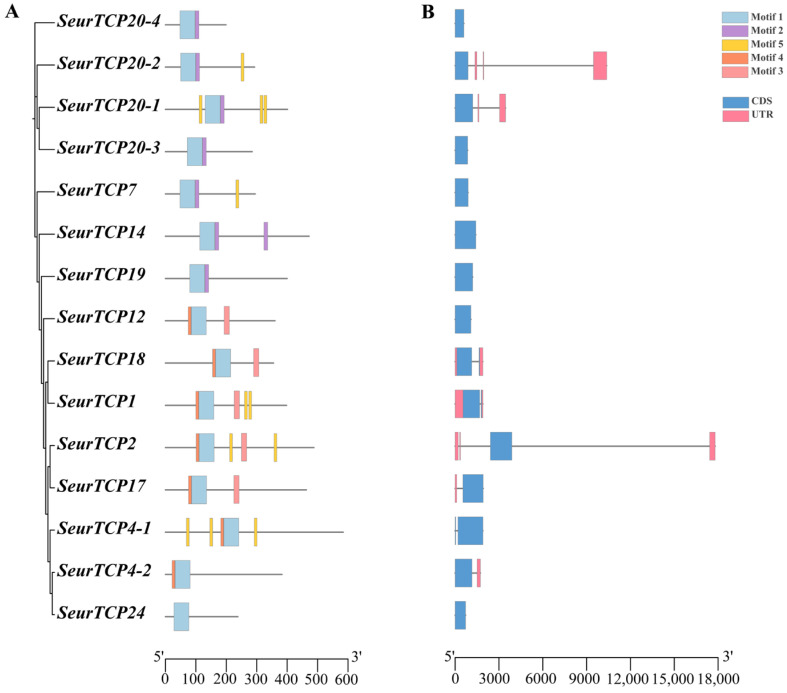
Phylogenetic clustering, conserved motifs, gene structures, and predicted 3D models of SeurTCPs. (**A**) Phylogenetic relationships and the distribution of conserved motifs in SeurTCP proteins. Different colored boxes correspond to specific Motifs (Motifs 1–5). (**B**) Exon-intron structures of SeurTCP genes. Blue boxes indicate coding sequences (CDS), pink boxes represent untranslated regions (UTRs), and black lines represent introns. The scale bar at the bottom can be used to estimate the length of the genes.

To explore the structural features of SeurTCPs, conserved motif and gene structure analyses were conducted. Conserved motif analysis identified five core motifs (Motif 1–5). The composition and arrangement of motifs displayed clear subfamily-specific patterns: most SeurTCPs contained 2–4 motifs, with Motif 1 present in all members, reflecting high conservation; in contrast, Motifs 3, 4, and 5 were restricted to specific clades, highlighting structural differences among subfamilies (Figure 3A).

Protein domain analysis showed that all SeurTCPs contain the typical TCP core domain (TCP superfamily), mainly distributed from the N-terminal to the mid-region of the protein, with similar arrangement patterns within the same subfamily. Some members (e.g., *SeurTCP12* and *SeurTCP2*) exhibited slight variations in domain conservation, suggesting potential functional diversification at the protein level (Figure 3B).

Gene structure analysis revealed differences at the genomic level. Most members exhibited relatively simple structures with a single exon, consistent with typical characteristics of the TCP family. A few genes (e.g., *SeurTCP2*, *SeurTCP20-2*) contained longer untranslated regions (UTRs), and gene lengths varied. Such structural differences may be associated with transcriptional regulation or functional divergence (Figure S3).

### 2.4. Secondary and Tertiary Structure Features of SeurTCPs

Secondary structure prediction indicated that all SeurTCP proteins are composed of α-helices (Hh), extended strands (Ee), and random coils (Cc). Random coils were the most abundant, accounting for 50.28–71.49% of the protein; α-helices ranged from 11.23% to 36.39%, while extended strands were the least represented, ranging from 11.23% to 23.50% (Table S3).

Based on tertiary structure predictions and phylogenetic grouping, SeurTCP proteins retained the core TCP structural features but showed certain structural differences among subfamilies (Figure S4). PCF subfamily members primarily consist of α-helices, with relatively compact overall conformations, and a proportion of random coil regions distributed on the periphery. Differences in the proportion of helices and flexible regions were observed among members. CYC/TB1 subfamily members (e.g., *SeurTCP1*, *SeurTCP12*, and *SeurTCP18*) exhibited high structural consistency, with relatively high α-helix content and stable overall conformations, while random coils were mainly located at the protein termini. In contrast, CIN subfamily members displayed greater structural variability; some (e.g., *SeurTCP4-2* and *SeurTCP17*) contained higher proportions of random coils, while others had relatively more α-helical structures. These structural differences suggest subfamily-specific conformational divergence, which may relate to functional differentiation.

### 2.5. Cis-Acting Elements in SeurTCP Promoters and Subfamily Differences

*Cis*-acting elements in the promoter regions (2000 bp upstream of the transcription start site) of the 15 SeurTCP genes were identified. All promoters contained basic elements such as TATA-box and CAAT-box. A total of 551 functional elements were detected and categorized into four groups: stress-responsive, light-responsive, hormone-responsive, and development-related elements. Stress-responsive elements were the most abundant (232), followed by light-responsive (145) and hormone-responsive elements (101), whereas development-related elements were relatively fewer (33) (Table S4).

**Figure 4:**
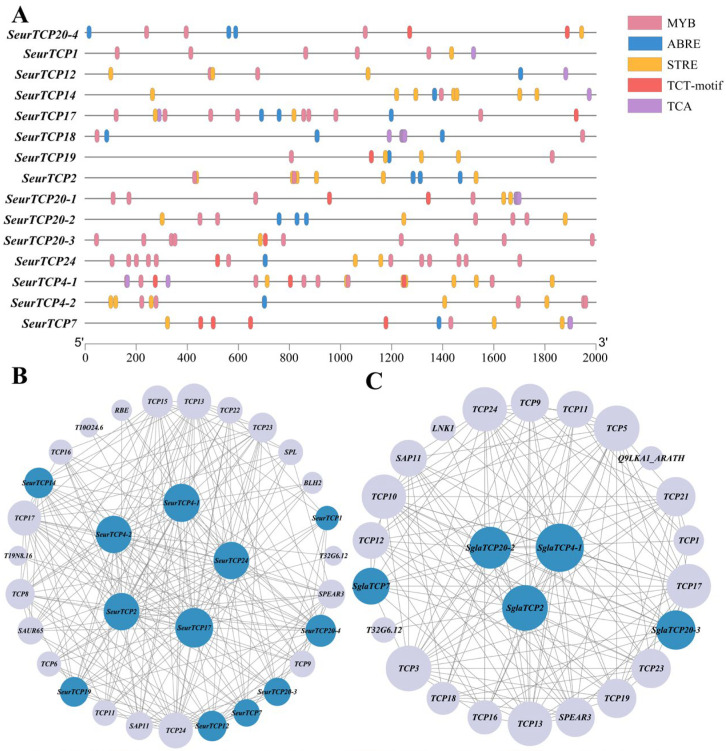
*Cis*-acting elements prediction and protein–protein interaction (PPI) networks of SeurTCPs. (**A**) Distribution of predicted *cis*-acting regulatory elements in the 2000 bp upstream promoter regions of *SeurTCP* genes. Different colored ovals represent distinct functional motifs (e.g., MYB, ABRE, STRE, TCT-motif, and TCA-element). (**B**) PPI networks associated with SeurTCPs based on orthologous mapping. Nodes represent proteins, and edges represent predicted functional associations or interactions. (**C**) PPI networks associated with SglaTCPs based on orthologous mapping. Nodes represent proteins, and edges represent predicted functional associations or interactions.

To compare regulatory characteristics among subfamilies, the distribution of typical *cis*-elements, including MYB, ABRE, STRE, TCT-motif, and TCA elements, was analyzed (Figure 4A). Results indicated clear differences in element composition and distribution among subfamilies. PCF subfamily promoters contained relatively diverse elements, with most members simultaneously possessing MYB, STRE, and ABRE elements, and enriched in TCT-motifs. CYC/TB1 subfamily promoters were simpler, lacking TCT-motifs, and primarily contained MYB and STRE elements, with some members also possessing TCA elements. CIN subfamily promoters contained more elements overall, with a dense distribution of MYB and STRE elements, and a widespread presence of ABRE and TCT-motifs.

### 2.6. Protein–Protein Interaction Network Analysis of SeurTCPs

To investigate the potential interaction relationships of SeurTCPs, a protein–protein interaction (PPI) network was constructed based on the STRING database. The network displayed a relatively centralized topology, with SeurTCP2, SeurTCP4-1, SeurTCP4-2, SeurTCP17, and SeurTCP24 located at the network center. These proteins showed higher degrees than other members in the predicted network and were therefore considered putative hub proteins. The core nodes formed relatively tight interaction clusters and were extensively connected with other members, including SeurTCP1, SeurTCP7, SeurTCP20-4, and SeurTCP20-3 (Figure 4B).

Comparative analysis of the TCP interaction network in the closely related species *Su. glauca* revealed a similarly centralized network. SglaTCP2, SglaTCP4-1, and SglaTCP20-2 were identified as major hub nodes, interacting with SglaTCP7, SglaTCP20-3, and several associated proteins such as SPL and SAP11 (Figure 4C). Overall, the TCP interaction networks of the two species exhibited similar global structures, though the composition of core nodes differed. Notably, CIN subfamily members (e.g., TCP2/4-type) were positioned at the network center in both species, suggesting that they may play a critical role in the TCP family interaction network.

### 2.7. Expression Patterns of SeurTCPs Under Salt Stress

**Figure 5:**
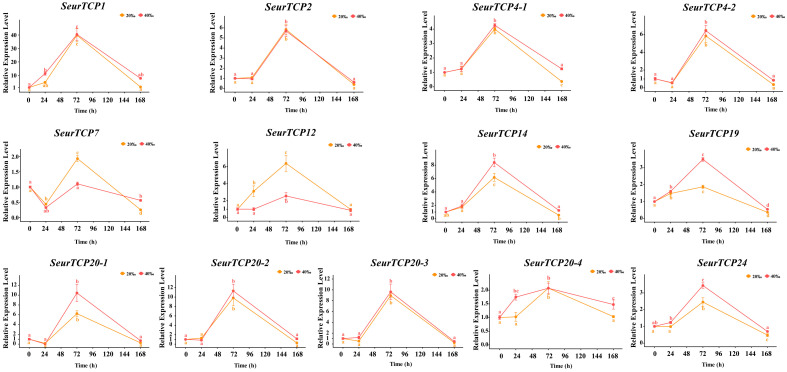
Expression profiles of SeurTCP genes in leaves under salinity stress. Relative expression levels of SeurTCP genes were determined by qRT-PCR in leaves of *S. europaea* subjected to 20‰ and 40‰ salinity treatments for 0, 24, 72, and 168 h. Expression levels were normalized to the reference gene and calculated using the 2^−ΔΔCt^ method. Data are presented as mean ± SD (*n* = 3). Different lowercase letters indicate significant differences among treatments according to Tukey’s HSD multiple range test (*p* < 0.05).

To investigate the transcriptional responses of SeurTCP genes to salinity treatment, qRT-PCR analysis was performed on representative TCP family members in leaves subjected to 20‰ and 40‰ salinity treatments, while expression patterns in roots are provided as supplementary data (Figure 5 and Figure S5). Two-way ANOVA showed that exposure time significantly affected the expression levels of most examined SeurTCP genes in both tissues (Table S5).

Members of the PCF subfamily generally exhibited pronounced changes in transcript abundance under salinity treatment. *SeurTCP1*, *SeurTCP19*, and *SeurTCP20-1/20-3* were markedly induced during the treatment period, with most genes reaching peak expression at 72 h, particularly under 40‰ salinity. *SeurTCP7* and *SeurTCP14* also showed significant transcriptional activation at intermediate stages of stress exposure. In the CYC/TB1 subfamily, *SeurTCP12* exhibited salt-responsive expression changes, whereas the remaining members showed relatively moderate changes. CIN subfamily genes, including *SeurTCP2*, *SeurTCP4-1*, *SeurTCP4-2*, and *SeurTCP24*, exhibited diverse expression patterns but were generally upregulated during salt treatment, with the highest transcript accumulation occurring around 72 h.

Expression profiles in roots showed partially similar trends to those observed in leaves, although the magnitude and timing of induction varied among genes. Overall, these results indicate that several SeurTCP genes are transcriptionally responsive to salinity treatment and may represent candidate genes for future functional studies. However, further experimental validation is required to determine their specific roles, if any, in salt stress responses or salt adaptation in *S. europaea*.

## 3. Discussion

TCP transcription factors, as plant-specific regulatory proteins, play crucial roles in plant growth, development, and responses to abiotic stresses, making them important subjects for investigating plant stress adaptation mechanisms [26]. *S. europaea*, as a typical extreme halophyte, serves as an ideal material for mining salt-tolerance genes and dissecting salt-adaptation mechanisms; however, its TCP gene family has not been systematically studied. In this study, 15 TCP family members were identified in the *S. europaea* genome and classified into three subfamilies: PCF, CIN, and CYC/TB1. The family size is comparable to diploid species such as grape (18 members) [27], strawberry (19 members) [28], and moso bamboo (16 members) [29], but is markedly smaller than that in polyploid species such as wheat (66 members) [30], switchgrass (42 members) [31], and Ma bamboo (60 members) [32]. These results indicate that the number of TCP family members varies among species and that genome ploidy may influence TCP family size.

Gene duplication is a key driver of gene family expansion and functional diversification [33]. In this study, SeurTCPs showed uneven chromosomal distribution, with relative enrichment on Chr6 and Chr9. Combined with the results of intra-genomic synteny analysis, multiple collinear gene pairs were observed between Chr6 and Chr1/Chr9, suggesting that this family may have undergone duplication-driven expansion. Similar distribution patterns have been reported in grape [27], maize [34], and cotton [35], implying that TCP family expansion patterns are somewhat conserved among different plants. It should be noted, however, that this study did not systematically classify the types of duplication (e.g., whole-genome duplication, segmental duplication, or tandem duplication), and thus the precise expansion mechanisms remain to be further explored.

Phylogenetic analysis and protein sequence alignment indicated that SeurTCPs can be divided into two major classes, Class I and Class II, and further into three subfamilies: PCF, CIN, and CYC/TB1. This classification framework is largely consistent with that in *Ar. thaliana* and other species [36]. Meanwhile, the PCF subfamily contains relatively more members, with some forming subgroups (e.g., SeurTCP20-1/2/3), indicating that this subfamily may have undergone local expansion during species divergence.

Gene structure analysis revealed that the majority of SeurTCPs possess a typical intronless structure with a single exon. This feature is highly consistent with TCP family characteristics in model plants and most angiosperms, reflecting strong structural conservation. Previous studies have shown that intron patterns in TCP genes are generally conserved in *Arabidopsis* and land plants, and TCP genes in melon also exhibit few introns or single-exon structures [37,38]. The absence of introns can significantly shorten mRNA processing time, thereby enabling rapid transcriptional and translational responses under stress conditions. This structural feature may provide a molecular basis for the rapid activation of defense mechanisms in *S. europaea* under extreme saline environments [39]. At the protein level, all SeurTCPs contain the conserved TCP domain and exhibit subfamily-specific differences in secondary and tertiary structures. Members of the PCF and CYC/TB1 subfamilies display relatively stable structural conformations, whereas the CIN subfamily shows greater structural variability. While these structural analyses confirm the overall conservation of the TCP domain and reveal subfamily-level structural differences, any specific functional implications of the identified motifs or 3D structural models cannot be inferred from the present computational data alone and will require dedicated experimental validation.

*Cis*-acting element analysis revealed that the promoter regions of SeurTCPs contain a variety of stress-responsive and hormone-responsive elements, with STRE and ABRE being frequently detected. Previous studies have shown that AREB/ABF-type bZIP factors act as central regulators of ABA-responsive gene expression during water-deficit and salt-related stress responses [40]. Furthermore, WRKY TFs have also been shown to regulate plant stress resistance via ABA-related pathways [41]. Multiple TCP transcription factors also participate in stress regulation via ABA-dependent pathways. For example, *TCP14/15* in *Arabidopsis* have been reported to regulate responses to salt and osmotic stress through ABA signaling pathways [36,42,43], and *TCP8* has also been shown to be involved in ABA-mediated drought stress responses [44]. SeurTCPs may participate in similar stress regulatory circuits—an hypothesis which requires experimental validation. Further analysis revealed that different subfamilies exhibit distinct *cis*-element compositions. The PCF subfamily contains a more diverse set of elements, the CIN subfamily is enriched in MYB-related elements, whereas the CYC/TB1 subfamily shows a relatively simpler composition. These differences may reflect functional divergence among subfamilies, although further functional studies are necessary to establish any causal links.

PPI network analysis indicated that some SeurTCPs (e.g., SeurTCP2 and TCP4-like members) occupy central positions in the network, exhibiting relatively high connectivity. Previous studies have shown that TCP proteins participate in interaction networks with other regulatory factors and homologous proteins through their conserved TCP domains, and that interaction patterns differ among subfamilies, providing a potential molecular basis for functional diversification within the family [36,37]. Nevertheless, given that this network was derived from in silico prediction using *Arabidopsis* orthologs, the inferred interactions should be interpreted with caution and require subsequent experimental confirmation to establish their biological significance.

qRT-PCR analysis showed that most SeurTCP genes responded transcriptionally to salinity treatment, although their expression patterns differed among subfamilies. Members of the PCF subfamily generally exhibited greater expression changes, whereas genes from the CYC/TB1 and CIN subfamilies displayed more diverse response patterns. Similar observations have been reported in cassava, where individual TCP genes showed distinct expression profiles under abiotic stress conditions [45]. Previous studies have shown that TCP transcription factors are involved not only in plant growth and development but also in responses to environmental cues, with distinct expression patterns often observed among TCP subfamilies [46]. Several SeurTCP genes maintained relatively high transcript abundance during salt treatment, suggesting their potential involvement in the adaptation of *S. europaea* to saline environments. However, because only four sampling points (0, 24, 72, and 168 h) were included, the early transcriptional responses of these genes remain to be characterized.

These salt-responsive expression patterns of SeurTCP genes should also be interpreted within the broader context of transcription factor-mediated abiotic stress regulation. Plant adaptation to salinity is rarely controlled by a single regulatory family; rather, multiple transcription factor modules jointly regulate hormone signaling, osmotic adjustment, antioxidant defense, and downstream stress-responsive gene expression. A classic example is *DREB1A/CBF3*, whose stress-inducible expression improved drought, salt, and freezing tolerance in Arabidopsis, demonstrating that a single transcription factor can activate a broad protective program under different abiotic stresses [47]. In rice, overexpression of the NAC transcription factor SNAC1 enhanced drought resistance and salt tolerance, partly through improved water-loss control and stress-related gene regulation [48]. Similarly, the bZIP transcription factors *AREB1* and *OsbZIP23* have been shown to regulate ABA-dependent stress signaling and to enhance drought and/or salinity tolerance [40,49]. These studies indicate that abiotic stress tolerance is mediated by interconnected transcriptional pathways. Therefore, although TCP proteins are the focus of the present study, SeurTCPs are likely to function as components of a larger regulatory network rather than as isolated regulators.

## 4. Materials and Methods

### 4.1. Plant Materials and Salinity Stress Treatments

*S. europaea* plants used in this study were collected from coastal mudflats in Yancheng, Jiangsu Province, China. Seedlings with uniform growth were selected and transferred to a hydroponic system containing Hoagland nutrient solution for a 3-day acclimation period under controlled growth conditions. After acclimation, seedlings were randomly assigned to three salinity treatments: 0‰ (control), 20‰ (moderate salinity stress), and 40‰ (high salinity stress). All treatment solutions were prepared by dissolving commercial sea salt (Instant Ocean) in Hoagland nutrient solution to achieve the desired salinity, as measured using a salinity refractometer. Four independent biological replicates were established for each treatment and control condition. At 24 h, 72 h, and 168 h after treatment initiation, root and leaf tissues were separately harvested, immediately frozen in liquid nitrogen, and stored at −80 °C until further analysis.

### 4.2. Genome-Wide Analysis of the TCP Gene Family in S. europaea

#### 4.2.1. Identification of TCP Genes in *S. europaea* and Related Species

TCP gene family members were identified based on the *S. europaea* genome data from our laboratory. The Hidden Markov Model (HMM) profile corresponding to the TCP conserved domain (TCP superfamily, PF03634) was downloaded from the Pfam database (http://pfam.xfam.org/, accessed on 24 December 2025) and employed as a query to scan the *S. europaea* protein database using HMMER (v3.3.1) [50,51]. Concurrently, known TCP protein sequences from *Arabidopsis thaliana* and other selected Magnoliopsida species were retrieved from the NCBI database (https://www.ncbi.nlm.nih.gov/protein/, accessed on 25 December 2025) and used as query sequences for BLASTp (v2.17.0) alignment against the *S. europaea* proteome (E-value ≤ 1 × 10^−5^). Details of the query sequences are shown in Table S1. The intersecting sequences identified by both HMMER and BLASTp approaches were retained and designated as SeurTCP genes. To facilitate comparative evolutionary analyses, an identical bioinformatic pipeline was applied to identify TCP genes in *Suaeda glauca* (WGS038631) [52], *Beta vulgaris* (GCA_026745355.1), *Amaranthus tricolor* (GCA_026212465.1), *Amaranthus hypochondriacus* (GCA_977020195.1), *Spinacia oleracea* (GCA_020520425.1), and *Bienertia sinuspersici* (GCA_044505025.1).

#### 4.2.2. Physicochemical Properties and Phylogenetic Analysis

The chromosomal coordinates of the identified SeurTCPs were extracted from the genomic GFF3 annotation files and physically mapped onto chromosomes using the Gene Location Visualizer module in TBtools-II (v2.472) [53,54]. The amino acid sequences of SeurTCP proteins were submitted to the ExPASy ProtParam online tool (https://web.expasy.org/protparam/, accessed on 7 January 2026) to predict their physicochemical properties, including amino acid sequence length, molecular weight (MW), theoretical isoelectric point (pI), and the grand average of hydropathicity (GRAVY) [55].

For phylogenetic analysis, multiple sequence alignments of the full-length TCP proteins from *S. europaea* and its six closely related species (*Su. glauca*, *Be. vulgaris*, *Am. tricolor*, *Sp. oleracea*, *Am. hypochondriacus*, and *Bi. sinuspersici*) were first performed using MUSCLE (v5.1). Subsequently, based on the alignment results, a Maximum Likelihood (ML) phylogenetic tree was constructed using FastTree (v2.1.11), and the generated evolutionary tree was visualized and annotated using the iTOL platform (https://itol.embl.de/, accessed on 9 January 2026) for visual annotation [13,56].

The exon-intron gene structural patterns of SeurTCPs were analyzed via the GSDS server. Conserved motifs in the protein sequences were predicted using the MEME Suite (https://meme-suite.org/meme/tools/meme, accessed on 15 January 2026), with the maximum number of motifs set to 5 and a motif width of 6–50 amino acids [57]. Conserved domains were verified using the NCBI Batch CD-Search tool (https://www.ncbi.nlm.nih.gov/Structure/bwrpsb/bwrpsb.cgi, accessed on 15 January 2026), and the integrated visualization of the phylogenetic tree, gene structures, conserved motifs, and domains was carried out using TBtools-II [58]. Finally, the secondary structural elements of SeurTCP proteins (including α-helices, β-sheets, and random coils) were predicted using the SOPMA tool (https://npsa.lyon.inserm.fr/, accessed on 17 January 2026), and their three-dimensional (3D) homology modeling was generated using the SWISS-MODEL server (https://swissmodel.expasy.org/interactive, accessed on 20 January 2026).

#### 4.2.3. Protein Interaction Network, Cis-Acting Elements, and Synteny Analysis

The SeurTCP amino acid sequences were submitted to the STRING database (https://cn.string-db.org/, accessed on 2 February 2026) to predict potential–protein interaction relationships, using *Ar. thaliana* as the reference organism with a minimum required interaction score of 0.4. The network was visualized in Cytoscape (v3.10.2) [59]. For *cis*-acting element analysis, the 2000 bp upstream sequence from the start codon (ATG) of each SeurTCP gene was extracted and submitted to the PlantCARE database to identify elements associated with stress, hormone signaling, and plant development [60]. Finally, genome-wide synteny analysis among *S. europaea* and the four related species (*Su. glauca*, *Be. vulgaris*, *Am. hypochondriacus*, and *Am. tricolor*) was performed using the MCScanX module integrated into TBtools-II to identify syntenic gene pairs and construct multi-species collinearity maps.

### 4.3. RNA Extraction and qRT-PCR Analysis of SeurTCP Genes

Total RNA was extracted from the root and leaf of each treatment group using FreeZol Reagent (Vazyme, R711-01, Nanjing, China). RNA concentration and purity were assessed using a NanoDrop 2000 spectrophotometer (Thermo Fisher Scientific, Waltham, MA, USA), and integrity was verified by 1% agarose gel electrophoresis. High-quality total RNA was reverse-transcribed into cDNA using the HiScript III RT SuperMix (Vazyme, R323, Nanjing, China). Gene-specific primers were designed using Primer Premier 6.0, and the *S. europaea* actin gene was utilized as the internal reference for normalization (primer sequences are detailed in Supplementary Table S2) [61]. qRT-PCR assays were performed on a QuantStudio 3 Real-Time PCR System (Thermo Fisher Scientific, Waltham, MA, USA) using the ChamQ SYBR Color qPCR Master Mix (Vazyme, Q411, Nanjing, China). Each sample comprised three biological replicates and three technical replicates. Relative gene expression levels were calculated using the 2^−ΔΔCt^ method [62]. All statistical analyses were performed using IBM SPSS v27 software. The homogeneity of variances across the treatment groups (salinity levels × exposure times) was evaluated for each target gene using Levene’s test based on the median. Levene’s test confirmed that the variances were homogeneous (*p* > 0.05). Subsequently, a two-way analysis of variance (ANOVA) was conducted for each gene to assess the main effects of salinity and exposure time, as well as their interaction. Post hoc pairwise comparisons were performed using Tukey’s HSD test, with statistical significance set at *p* < 0.05. Data are presented as means ± standard deviation (SD). Graphs were generated with GraphPad Prism 9 software.

## 5. Conclusions

This study systematically identified and characterized the TCP gene family in *S. europaea*, classifying 15 members into the PCF, CIN, and CYC/TB1 subfamilies. SeurTCPs exhibit high structural conservation alongside clear subfamily-specific features, including motif composition, structural variation, and *cis*-element distribution, suggesting functional diversification. Network analysis identified CIN and PCF members as potential regulatory hubs, while qRT-PCR confirmed their organ-specific expression and differential responses to salinity. Together, these results indicate that SeurTCPs integrate conserved functions with subfamily-specific regulation, contributing to salt stress adaptation in *S. europaea* and providing candidate genes for improving crop salt tolerance.

## Figures and Tables

**Table 1:** Physicochemical properties of the SeurTCPs.

Gene ID	Gene Name	Amino Acid Length (aa)	Molecular Weights (Da)	Isoelectric Point (pI)	Instability Index	Aliphatic Index	Grand Average of Hydropathicity (GRAVY)
*Seuropaea000175*	*SeurTCP12*	360	40,706.13	5.49	35.92	55.03	−1.251
*Seuropaea000906*	*SeurTCP20-1*	401	43,836.97	6.96	50.41	59.15	−1.001
*Seuropaea006178*	*SeurTCP20-4*	199	21,960.21	6.70	52.39	60.80	−0.708
*Seuropaea008339*	*SeurTCP4-1*	584	63,692.31	6.46	61.74	55.00	−0.903
*Seuropaea013853*	*SeurTCP18*	355	40,172.16	6.54	46.70	57.66	−1.010
*Seuropaea013923*	*SeurTCP17*	566	50,760.04	6.53	57.58	51.64	−0.957
*Seuropaea014067*	*SeurTCP14*	463	49,849.99	6.97	54.07	53.28	−0.796
*Seuropaea014482*	*SeurTCP20-2*	293	31,255.30	7.92	44.51	70.96	−0.615
*Seuropaea015266*	*SeurTCP19*	400	41,857.71	5.66	52.65	60.95	−0.720
*Seuropaea015901*	*SeurTCP7*	295	30,980.31	9.46	48.51	67.97	−0.496
*Seuropaea016107*	*SeurTCP1*	398	46,226.41	6.42	63.15	58.34	−1.154
*Seuropaea020824*	*SeurTCP2*	488	52,686.89	9.16	50.26	49.57	−1.034
*Seuropaea021440*	*SeurTCP24*	238	27,524.96	6.23	56.17	67.18	−0.692
*Seuropaea021745*	*SeurTCP4-2*	383	40,683.54	6.41	47.83	56.29	−0.652
*Seuropaea022434*	*SeurTCP20-3*	285	31,476.89	9.38	49.79	65.02	−0.815

## Data Availability

Data are contained within the article and Supplementary Materials.

## Supplementary Materials

The following supporting information can be downloaded at: https://www.mdpi.com/article/10.3390/ijms27125514/s1.
