# Supplementary material for: Genome-Wide Characterization of TCP Transcription Factors in Common Glasswort (Salicornia europaea) and Their Expression Analysis Under Salt Stress
Source: Int J Mol Sci. 2026 Jun 18;27(12):5514. doi: 10.3390/ijms27125514 (PMC13299481; doi:10.3390/ijms27125514)
Supplement: Supplementary file 1 [file ijms-27-05514-s001.zip › Supplementary Figures S1-S5.pdf]

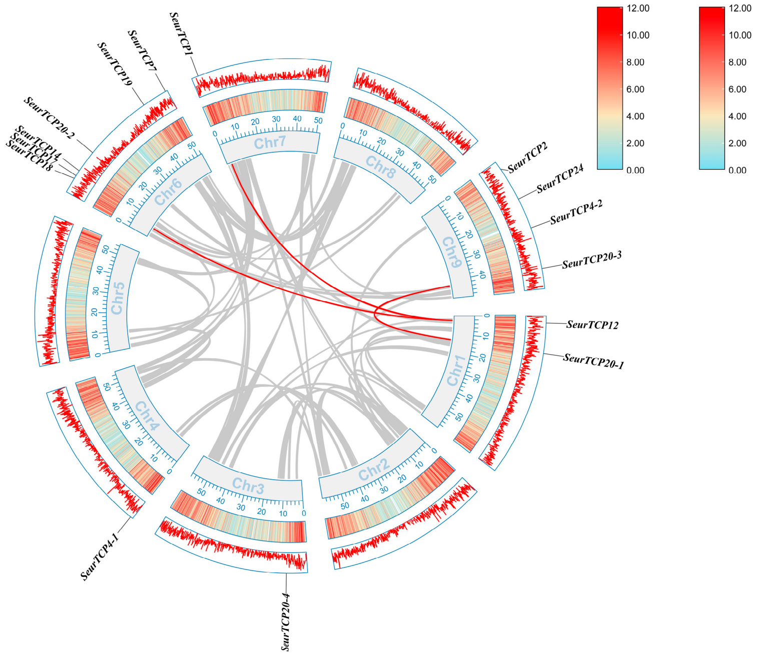

Figure S1. Chromosomal distribution and intra-species synteny of SeurTCP genes. Outer circles show the mapping positions of SeurTCPs and chromosomal gene density (heatmaps and line plots). Central grey lines indicate genome-wide collinear blocks, while red lines highlight segmentally duplicated SeurTCP gene pairs.

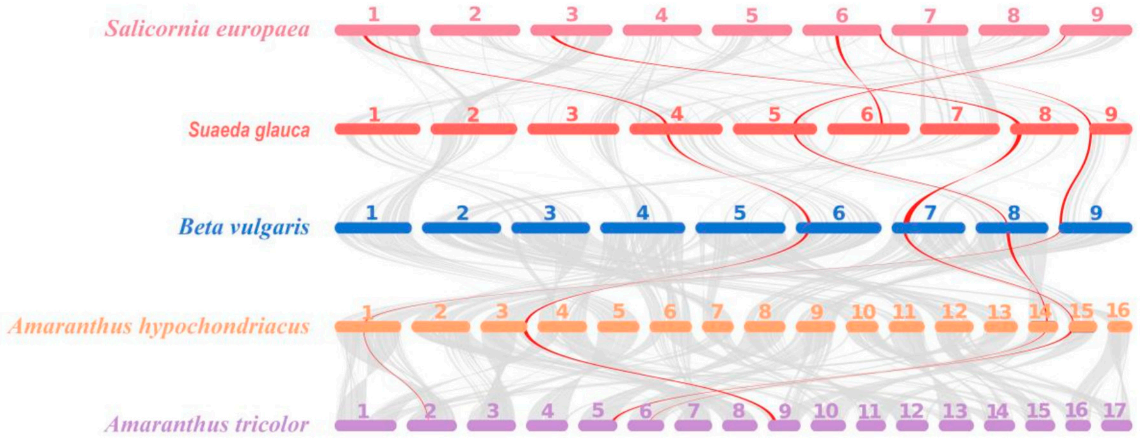

Figure S2. Collinearity analysis of TCP genes among *Salicornia europaea*, *Suaeda glauca*, *Beta vulgaris*, *Amaranthus hypochondriacus*, and *Amaranthus tricolor*. The grey lines in the background represent the collinear blocks across the whole genomes, while the red lines highlight the orthologous TCP gene pairs among the diverse species.

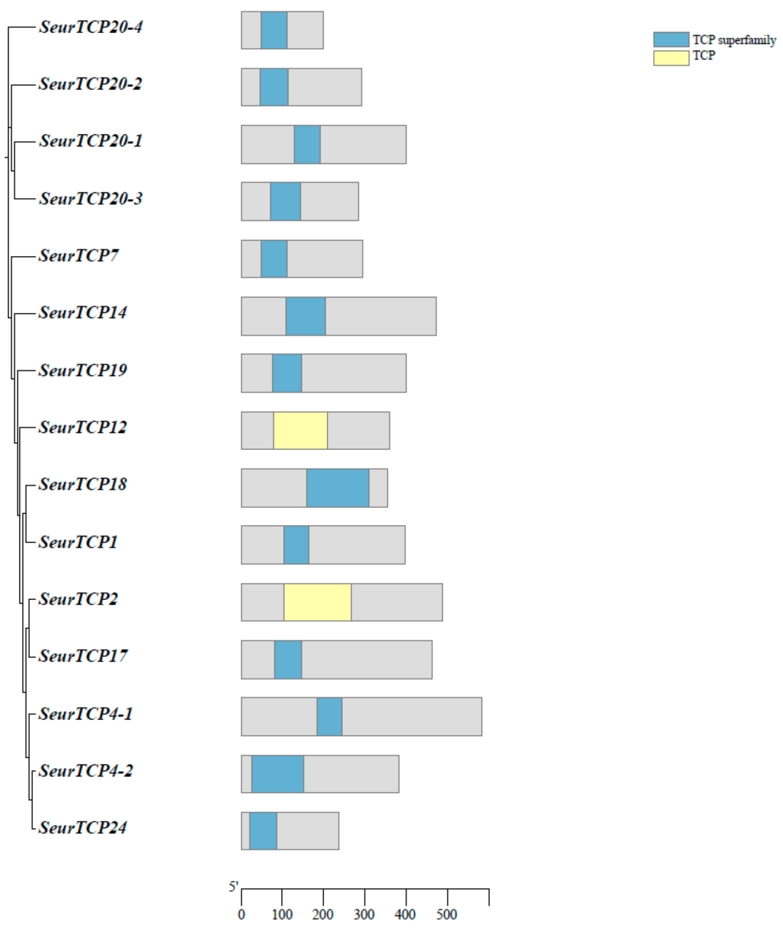

Figure S3. Conserved domain architecture of SeurTCP proteins. Blue and yellow boxes denote the predicted TCP superfamily and TCP domains, respectively. The bottom scale bar indicates the protein length in amino acids.

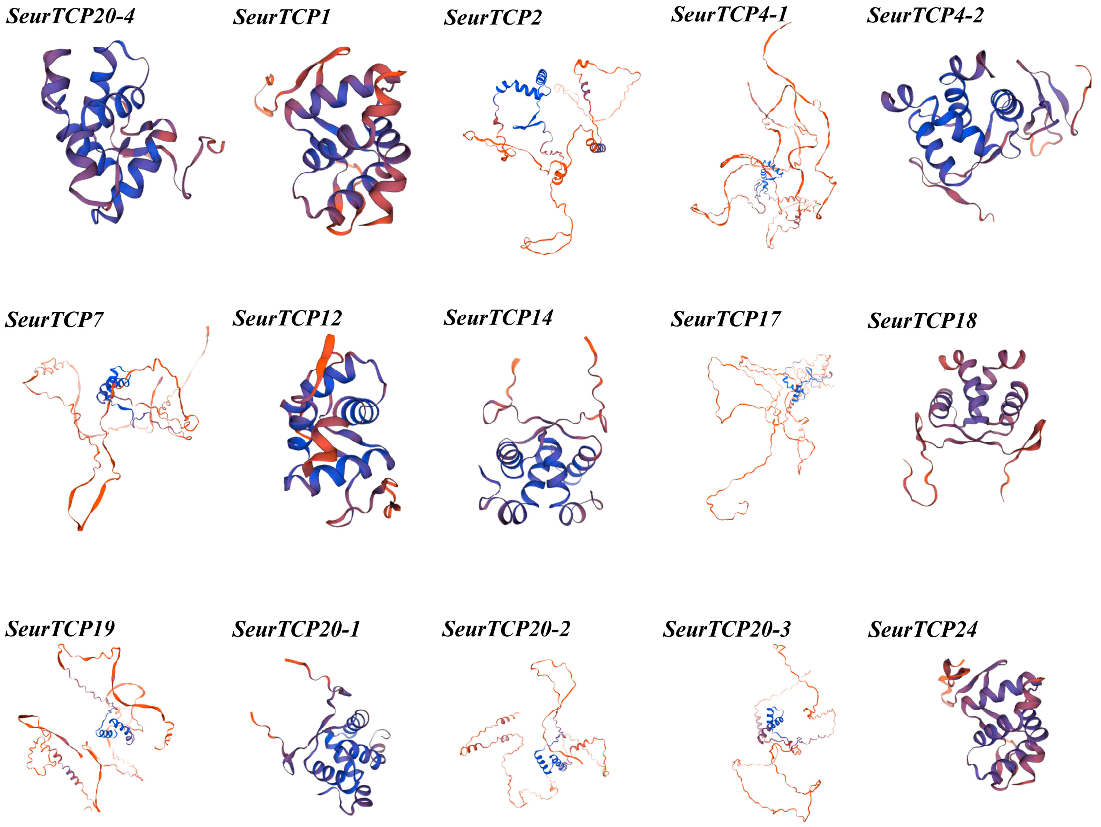

Figure S4. Predicted three-dimensional (3D) spatial structures of SeurTCP proteins generated by homology modeling.

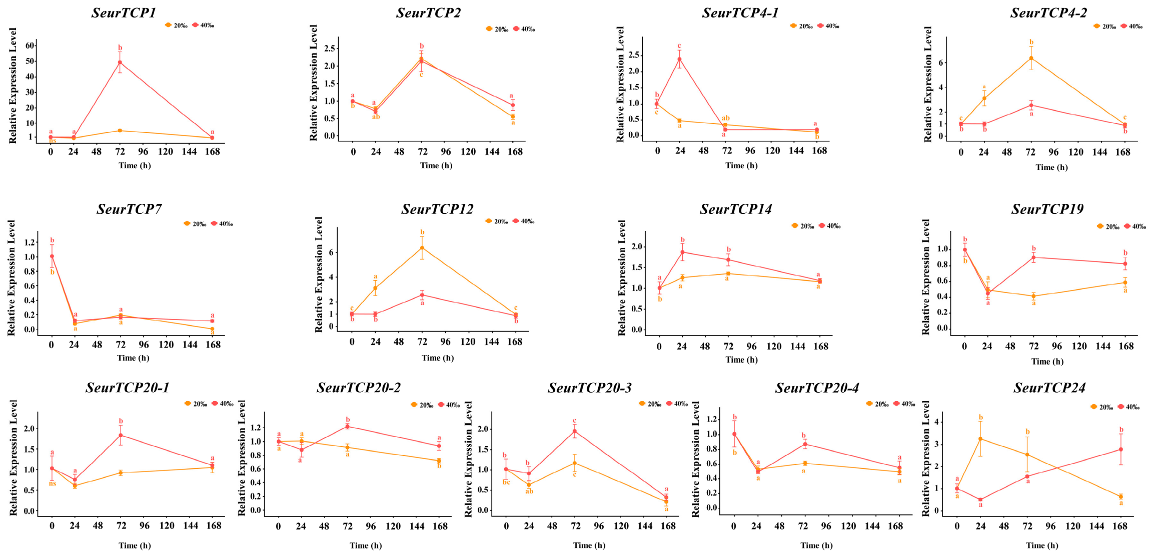

Figure S5. Relative expression levels of SeurTCP genes in roots of Salicornia europaea under 20‰ and 40‰ salinity treatments at different time points. Data are presented as mean ± SD (n = 3). Different lowercase letters indicate significant differences ($p < 0.05$).
